# Benchmarking ChatGPT-3.5 and OpenAI o3 Against Clinical Pharmacists: Preliminary Insights into Clinical Accuracy, Sensitivity, and Specificity in Pharmacy MCQs

**DOI:** 10.3390/healthcare13141751

**Published:** 2025-07-19

**Authors:** Esraa M. Alsaudi, Sireen A. Shilbayeh, Rana K Abu-Farha

**Affiliations:** 1 Clinical Pharmacy and Therapeutics Department, Faculty of Pharmacy, Applied Science Private University, Amman P.O. Box 11937, Jordan; e.alsaudi@ju.edu.jo; 2 Department of Pharmacy Practice, College of Pharmacy, Princess Nourah bint Abdulrahman University, Riyadh 11671, Saudi Arabia; ssabdulrahim@pnu.edu.sa

**Keywords:** clinical accuracy, ChatGPT-3.5, OpenAI o3, pharmacists, reproducibility

## Abstract

**Objective:** This proof-of-concept study aimed to evaluate and compare the clinical performance of two AI language models (ChatGPT-3.5 and OpenAI o3) in answering clinical pharmacy multiple-choice questions (MCQs), benchmarked against responses from specialist clinical pharmacists in Jordan, including academic preceptors and hospital-based clinicians. **Methods:** A total of 60 clinical pharmacy MCQs were developed based on current guidelines across four therapeutic areas: cardiovascular, endocrine, infectious, and respiratory diseases. Each item was reviewed by academic and clinical experts and then pilot-tested with five pharmacists to determine clarity and difficulty. Two ChatGPT models—GPT-3.5 and OpenAI o3—were tested using a standardized prompt for each MCQ, entered in separate sessions to avoid memory retention. Their answers were classified as true/false positives or negatives and retested after two weeks to assess reproducibility. Simultaneously, 25 licensed pharmacists (primarily from one academic institution and several hospitals in Amman) completed the same MCQs using validated references (excluding AI tools). Accuracy, sensitivity, specificity, and Cohen’s Kappa were used to compare AI and human performance, with statistical analysis conducted using appropriate tests at a significance level of *p* ≤ 0.05. **Results:** OpenAI o3 achieved the highest accuracy (83.3%), sensitivity (90.0%), and specificity (70.0%), outperforming GPT-3.5 (70.0%, 77.5%, 55.0%) and pharmacists (69.7%, 77.0%, 55.0%). AI performance declined significantly with increasing question difficulty. OpenAI o3 showed the highest accuracy in the cardiovascular domain (93.3%), while GPT-3.5 performed best in infectious diseases (80.0%). Reproducibility was higher for GPT-3.5 (81.6%, κ = 0.556) than OpenAI o3 (76.7%, κ = 0.364). Over two test rounds, GPT-3.5’s accuracy remained stable, whereas OpenAI o3’s accuracy decreased from 83.3% to 70.0%, indicating some variability. **Conclusions:** OpenAI o3 shows strong promise as a clinical decision-support tool in pharmacy, especially for low- to moderate-difficulty questions. However, inconsistencies in reproducibility and limitations in complex cases highlight the importance of cautious, supervised integration alongside human expertise.

## 1. Introduction

The concept of clinical pharmacy emerged in the United States during the mid-1960s, marking a pivotal shift in the pharmacist’s role from mere dispensers of medication to active participants in patient-centered care [1]. Since then, rapid advancements in healthcare have expanded the responsibilities of clinical pharmacists. The increasing complexity of drug therapies, rising prescription volumes, and mounting administrative duties have created significant demands on pharmacy professionals [2]. These evolving challenges highlight the growing need for innovative, technology-driven solutions that can enhance efficiency and support pharmacists in delivering high-quality care [3].

One such innovation is the integration of artificial intelligence (AI) into healthcare systems. AI technologies offer promising tools for streamlining workflow, improving medication management, and ultimately enhancing patient outcomes. Among the most widely recognized AI applications is ChatGPT, a large language model developed by OpenAI and released in November 2022 [4]. In pharmacy practice, AI has shown potential in tasks such as medication reconciliation, identifying drug interactions, improving adherence, reducing costs, and addressing issues related to polypharmacy [5,6,7].

However, the clinical utility of AI models like ChatGPT depends heavily on the accuracy and relevance of the information they generate. These models are trained on large-scale datasets that include medical literature, drug databases, and clinical guidelines, which enables them to generate informed responses to medical queries, despite lacking actual clinical experience or reasoning [8]. Limitations in data quality, as well as the absence of integration with authoritative drug information resources—such as Lexicomp, Micromedex, and UpToDate—can result in errors, inconsistencies, and inadequate clinical reasoning [6,9,10]. Studies have highlighted that while AI can perform well in certain structured tasks, it often struggles with complex clinical questions that demand nuanced judgment [11,12].

Despite the growing interest in AI applications across healthcare, there remains a notable gap in empirical research evaluating how tools like AI tools perform in real-world pharmacy contexts. This study investigates the performance of two models developed by OpenAI—GPT-3.5 and the more recent OpenAI o3 model, by comparing their responses to those of licensed clinical pharmacists on a structured set of multiple-choice questions (MCQs) covering various therapeutic topics and difficulty levels. In this context, “clinical pharmacists” refers specifically to a sample of specialist pharmacists in Jordan, including academic preceptors and hospital-based practitioners, most of whom were based in Amman. The aim is not to explore AI as a replacement for pharmacists, but to assess its potential as a supportive tool in clinical decision-making and to highlight its current limitations.

## 2. Materials and Methods

### 2.1. Study Design and Clinical Cases Development

This study aimed to evaluate the performance of the latest free version of ChatGPT (GPT-3.5) and a subscription-based version (OpenAI o3, which was released in April 2025) in answering clinical pharmacy MCQs and compared their performance to that of specialist clinical pharmacists. A set of 60 MCQs was developed by the primary author, an academic clinical pharmacy practitioner, based on recent guidelines in four therapeutic areas: cardiovascular disease, endocrine disorders, infectious diseases, and respiratory conditions. These areas were chosen to cover a wide range of clinical pharmacy work, such as ensuring medication efficacy (picking the right treatments), safety (side effects), contraindications, and spotting interactions between different drugs.

The MCQs were designed to assess the critical aspects of clinical pharmacy practice that pharmacists are expected to handle in everyday practice. To ensure validity, the questions underwent independent review by two experts: an academic pharmacology specialist who also supervised the research, and a clinical pharmacy practitioner with academic and professional experience. Both reviewers evaluated the MCQs for clinical relevance, accuracy, and clarity, ensuring alignment with current practice standards. All MCQs were in English, which is the formal language for pharmacy education in Jordan, where the study took place.

Each MCQ had four different choices (A, B, C, and D), which may not necessarily contain the correct answer. While answering the questions, respondents could select “None” if they believed that none of the options provided were correct.

### 2.2. Pilot Testing and Difficulty Index Calculation

A pilot test was conducted with five clinical pharmacists to evaluate the difficulty and clarity of the MCQs. Based on their feedback, several questions were refined (Table 1). Also, the difficulty index (DI) of each question was calculated based on the proportion of correct responses from the pilot group. Questions were classified as “difficult” if the DI was ≤0.40, “average” if the DI was >0.40 and ≤0.80, and “easy” if the DI was >0.80.

### 2.3. AI Tool Selection, Interaction, and Testing

Two OpenAI models were selected to evaluate their performance in answering the 60 MCQs: the free version of ChatGPT (GPT-3.5) and the subscription-based OpenAI model “o3.” The same prompt was used for both AI models, as follows: “For the following clinical pharmacy MCQ, please select the most appropriate answer for each question. Please note that each question has only one correct answer. If you believe none of the options is correct, write “None” as your response.”

Each MCQ was entered separately into the AI tools using the “New Chat” function, with memory disabled, to prevent any memory retention between questions. This ensured that the interaction is independent and unaffected by previous responses. After each question was answered, a screenshot was obtained to capture the AI’s response, including the selected answer and any explanations provided. These screenshots served as a record for further analysis and comparison with the pharmacists’ responses.

The AI’s responses were categorized as follows:

- True Positive (TP): The AI selected the correct answer among the four choices where a correct response is available.
- False Negative (FN): The AI selected an incorrect answer (either an incorrect choice from the four options, or “None”), even though a correct response is available.
- True Negative (TN): The AI correctly selected “None” when no answer is deemed correct.
- False Positive (FP): The AI selected an incorrect answer from the four choices while no answer is deemed correct.

### 2.4. Evaluation of Clinical Pharmacists’ Performance

Pharmacists were recruited from the Faculty of Pharmacy at the University of Jordan, including academic preceptors, as well as from several hospitals in Amman to represent clinical practice settings. A convenience sampling method was employed. Twenty-five clinical pharmacists were invited using a convenience sampling method to complete the MCQs under supervised conditions. All 25 invited participants agreed to take part in the study. Invitations were disseminated through professional pharmacy networks and academic institutions in Jordan. All participants were licensed pharmacists holding either a Doctor of Pharmacy (PharmD) or a master’s degree in clinical pharmacy, with current professional experience as clinical pharmacists or academic preceptors involved in clinical training. Eligibility criteria included (1) currently practicing as a clinical pharmacist or academic preceptor, (2) holding a PharmD or MSc in Clinical Pharmacy, and (3) having a minimum of six months of clinical experience. There were no exclusion criteria other than failure to complete the full questionnaire. Clinical pharmacists were given the authority to answer questions using reliable knowledge sources, whether books or trusted online sources, with the emphasis that no question can be answered using any type of AI tools.

The exam link, hosted on Google Forms, was provided to the pharmacists, who were given two days to complete the exam. Upon completion, pharmacists received certificates of participation, but they were not compensated financially. No personal identifying information was collected to ensure participant anonymity. The pharmacists’ responses were categorized in the same manner as the AI responses (TP, TN, FN, FP). This enabled a direct and standardized comparison between the pharmacists and AI models.

### 2.5. AI Tools and Pharmacists Performance

The performance of ChatGPT tools and the pharmacists was assessed using accuracy, sensitivity, and specificity as follows:

- Accuracy: The proportion of questions answered correctly by both the AI tools and pharmacists. [Accuracy = TP + TN/(TP + TN + FP + FN)].
- Sensitivity: A measure of the ability of the AI (or a pharmacist) to correctly identify true positives (the correct answers) from all actual positives (questions where the correct answer is available). [Sensitivity = TP/(TP + FN)].
- Specificity: A measure of the ability of the AI (or a pharmacist) to correctly identify true negatives (when “None” is the correct answer) from all actual negatives (questions where no answer is correct). [Specificity = TN/(TN + FP)].
- The Cohen’s Kappa (κ) statistic was used to assess the agreement between the two AI tools. This statistic measures the level of agreement beyond chance, where values closer to 1 indicate strong agreement.

### 2.6. Reproducibility Assessment

To ensure the stability and reliability of the AI tools, a second round of testing was conducted two weeks after the initial evaluation. Each AI model was exposed to the same set of 60 MCQs using the identical prompt as before. The answers from both rounds were compared to evaluate the consistency of the responses.

Metrics for assessing reproducibility included

- Reproducibility Index: The proportion of identical answers and explanations between the two rounds for each AI tool.
- Intra-Rater Agreement: A second Cohen’s Kappa (κ) value was calculated to determine the level of agreement between the AI responses in both rounds.

### 2.7. Statistical Analysis

The data collected from the responses of both the AI tools and the pharmacists were analyzed using IBM SPSS Statistics Version 22 (IBM Corp, Armonk, NY, USA, 2013). For comparing diagnostic performance metrics between pharmacists and AI tools, a One-Sample t-test was used for normally distributed outcomes, while the One-Sample Wilcoxon Signed-Rank Test was applied for non-normally distributed outcomes. Pairwise agreement between the two AI models was assessed using Cohen’s Kappa. When evaluating the accuracy across different difficulty levels and therapeutic systems, Chi-square or Fisher’s Exact Test was used. Reproducibility between rounds was assessed using Cohen’s Kappa. For evaluating the change in accuracy between multiple test runs, the McNemar test was applied. A significant level of *p* ≤ 0.05 was considered to indicate statistically significant differences.

## 3. Results

In this study, a total of 60 clinical case-based MCQs were developed to evaluate AI and pharmacists’ performance in pharmacotherapeutic decision-making. These cases were evenly distributed across four major therapeutic systems: cardiovascular, endocrine, respiratory, and infectious diseases (15 questions each). Each case was categorized under one of four key clinical domains: efficacy, safety, contraindications, and drug interactions. The majority of questions assessed efficacy (*n* = 25, 41.7%) and safety (*n* = 22, 36.7%), while fewer addressed contraindications (*n* = 10, 16.7%) and drug interactions (*n* = 3, 5%).

The MCQs were categorized based on their DI into three levels: Easy (DI > 0.8), Average (DI = 0.4–0.8), and Difficult (DI < 0.4). Among the questions, 16 questions (*n* = 16, 26.7%) were classified as easy, while another 16 were difficult (*n* = 16, 26.7%). The remaining 28 questions (46.7%) fell into the average range. The cardiovascular system had the highest number of easy questions, while both the respiratory and infectious disease systems had the most difficult ones (6 each). Table 2 presents examples of the three easiest and three most difficult MCQs based on their DI, ranging from 1.0 (easiest) to 0.0 (most difficult).

The majority of participants were female (*n* = 23, 92%). Most held a PharmD degree (*n* = 15, 60%), and the remaining held a master’s degree (*n* = 10, 40%). Regarding professional roles, 19 (76%) were academics or clinical preceptors, and 6 (24%) were clinical pharmacists working in hospitals.

For the pharmacists, the number of TP ranged from 25 to 35, with a mean of 30.8 (SD = 2.7). The number of FN ranged from 5 to 15, with a mean of 9.2 (SD = 2.7). TN ranged from 6 to 18, with a mean of 11.0 (SD = 3.4), and FP ranged from 2 to 14, with a mean of 9.0 (SD = 3.4). On the other hand, for GPT-3.5, the number of TP was 31, TN was 11, FP was 9, and FN was 9, while for OpenAI o3, the number of TP was 36, TN was 14, FP was 6, and FN was 4.

Regarding the overall errors for both AI tools and clinical pharmacists, the GPT-3.5 group had 18 errors (9 FP and 9 FN), the OpenAI o3 group had 10 errors (6 FP and 4 FN), and clinical pharmacists, on average, had 18.2 errors (9.0 FP and 9.2 FN).

**Figure 1:**
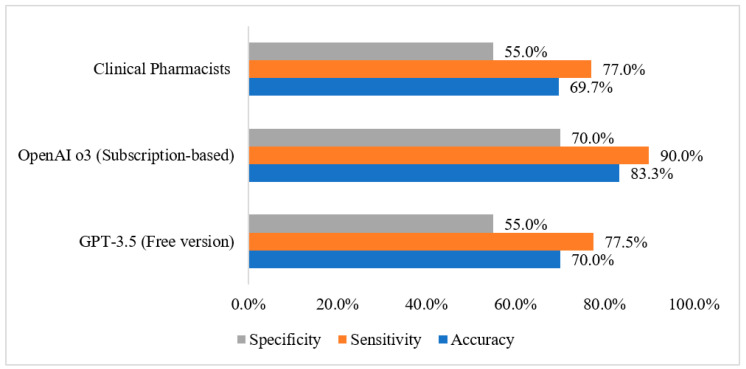
Diagnostic performance metrics by group.

The diagnostic performance metrics for each group are summarized in Figure 1. For the GPT-3.5, the accuracy was 70.0%, sensitivity reached 77.5%, and specificity was 55.0%. The OpenAI o3 showed improved performance with an accuracy of 83.3%, sensitivity of 90.0%, and specificity of 70.0%. In comparison, the clinical pharmacists’ group had an accuracy of 69.7%, sensitivity of 77.0%, and specificity of 55.0%.

Diagnostic performance metrics were compared between clinical pharmacists and the two AI tools, as shown in Table 3. For accuracy, there was no significant difference between pharmacists and GPT-3.5 (*p* = 0.852). However, pharmacists performed significantly lower than OpenAI o3, with a *p*-value of less than 0.001. In terms of sensitivity, there was no significant difference between pharmacists and GPT-3.5 (*p* = 0.718), but pharmacists had significantly lower sensitivity compared to OpenAI o3 (*p* < 0.001). As for specificity, no significant difference was found between pharmacists and GPT-3.5 (*p* = 0.965), but pharmacists were significantly lower than OpenAI o3 in specificity (*p* = 0.001).

When evaluating each AI tool’s performance across varying difficulty levels (Table 4), a significant decline in accuracy was observed as question difficulty increased. For GPT-3.5, correct responses dropped from 87.5% on easy items to 78.6% on average and 37.5% on difficult items (*p* = 0.007). Similarly, OpenAI o3 showed a decrease from 93.8% (easy) to 92.9% (average) and 56.3% (difficult), with a statistically significant difference (*p* = 0.006).

When analyzing performance across therapeutic systems, both AI tools showed variation in accuracy depending on the system (Table 5). GPT-3.5 achieved its highest accuracy in infectious diseases (80.0%) and lowest in cardiovascular (60.0%), while OpenAI o3 performed best in cardiovascular (93.3%) and maintained high accuracy in respiratory and infectious diseases (both 86.7%). Despite these trends, the differences across therapeutic areas were not statistically significant for either model (*p* = 0.782 for GPT-3.5, *p* = 0.357 for OpenAI o3).

The pairwise agreement between GPT-3.5 and OpenAI o3, as measured by Cohen’s Kappa, was 0.364 with a statistically significant *p*-value of 0.002, indicating a fair level of agreement and a significant difference in their response patterns. Among the 60 questions, 14 exhibited disagreements between the AI models (GPT-3.5 and OpenAI o3) (Table 6).

When evaluating reproducibility between two rounds conducted two weeks apart (Table 7), GPT-3.5 showed higher consistency, providing identical answers for 49 out of 60 questions (81.6%) with a Cohen’s Kappa of 0.556 (*p* < 0.001), indicating moderate agreement. On the other hand, OpenAI o3 produced identical responses in 46 out of 60 questions (76.7%) with a Cohen’s Kappa of 0.364 (*p* = 0.002), reflecting fair agreement.

Finally, when evaluating the accuracy of the AI tools across two test runs (Table 8), GPT-3.5 showed a slight improvement in accuracy from 70.0% in the first run (42 correct answers) to 71.6% in the second run (43 correct answers). The *p*-value for this change was 1.000, indicating no significant difference. In contrast, OpenAI o3 started with a higher accuracy of 83.3% in the first run (50 correct answers), but its accuracy dropped to 70.0% in the second run (42 correct answers). This change, with 11 answers changing from correct to incorrect and 3 from incorrect to correct, resulted in a *p*-value of 0.057, which suggests a slight trend toward a change, but it did not reach statistical significance.

## 4. Discussion

As hospitals start using AI tools, it is important to understand how their performance compares to that of clinical pharmacists. This study aims to measure the performance of the free ChatGPT (GPT-3.5) as well as the subscription-based model (OpenAI o3) compared to the performance of 25 clinical pharmacists in answering 60 clinical case questions distributed equally across four medical systems.

The pilot group’s reported DI showed a balanced distribution, with roughly half (47%) of the questions falling into the average range, 27% classified as easy, and another 27% are difficult. This is a good sign, as it means that the assessment questions were comprehensive and considerately prepared. Researchers currently agree that including questions of varying difficulty can help recognize how well respondents understand different subject areas [13].

This study showed that GPT-3.5 performed at a level comparable to clinical pharmacists, with both achieving an overall accuracy of 70%. In contrast, the subscription-based OpenAI o3 demonstrated significantly better performance, achieving 83.3% accuracy, 90.0% sensitivity, and 70.0% specificity. These differences were statistically significant, highlighting that more advanced AI models can outperform both earlier AI versions and trained healthcare professionals in certain decision-making capabilities.

It is important to acknowledge the phenomenon of “hallucinations” in AI language models, where the system may confidently generate incorrect or fabricated answers despite sounding plausible [14]. This issue was apparent in our findings, as even the more advanced OpenAI o3 occasionally provided inaccurate responses, especially on complex clinical questions. Such hallucinations emphasize the necessity of human supervision when employing AI in clinical decision-making, ensuring that AI outputs are carefully verified before application in patient care.

The findings of our study were comparable to a study conducted by Albogami et al. who assessed the differences in performance between three different AI tools (GPT-3, GPT-3.5, and GPT-4) and licensed clinical pharmacists in answering different real clinical cases [15]. In their study, researchers showed that the most advanced GPT-4, which is a subscription-based tool, achieved an accuracy of 64.3%, which was comparable to that licensed pharmacists, while it was superior to that of GPT-3, GPT-3.5 when answering the assessment cases. Both GPT-4 and pharmacists provided 95% safe responses [15]. These results, alongside ours, suggest that the more advanced AI generations have a better performance in supporting clinical decision-making.

Likewise, our findings were comparable to another study that investigated the effectiveness of GPT-3.5 and GPT-4 models in answering the Taiwan National Pharmacist Licensing Examination. The models were tested on basic and clinical subjects. The results showed that GPT-4 had a higher accuracy rate of 72.9%, outperforming GPT-3.5 in basic subjects. However, in clinical subjects, only minor differences were observed, with GPT-4 outperforming GPT-3.5 in calculation and situational questions [16].

Conversely, our findings were inconsistent with a previous study that evaluated ChatGPT’s performance on a fourth-year pharmacy student exam at Chiang Mai University (Thailand). The exam consisted of 16 multiple-choice questions and 2 short-answer questions. ChatGPT provided 44% correct responses, while students provided 66%. The study found that AI has limitations when faced with practical scenarios, highlighting the need for more exploration and collaboration [17].

Overall, our findings suggest the notion that as AI models continue to develop, their potential performance in addressing decision-support roles exceed that of human healthcare providers. However, one of the most significant challenges associated with using advanced models like OpenAI o3 is that they require a paid subscription, limiting access to them to individuals or institutions with limited financial capacity. This poses a real obstacle for institutions with limited resources, not only in low-income countries but also in underfunded hospitals and clinics within high-income countries. The varying financial models, such as flat-rate subscriptions or pay-per-use fees, further complicate access. Consequently, these barriers contribute to unequal opportunities and raise important ethical questions about fairness and digital equity in healthcare and medical education.

In a striking manner, this study concludes an important trend that accuracy decreases as question difficulty increases, this trend is valid for both AI tools in a statistically significant difference (*p* < 0.05). This suggest that despite having powerful capabilities, OpenAI o3 made errors on challenging medical questions, which is why using this tool should be guided by humans in complex healthcare circumstances.

This study also has measured the accuracy difference between both AI tools across therapeutic systems. OpenAI o3 excelled in every domain and was especially effective in the cardiovascular system. Although no statistically significant difference was found (*p* > 0.05), this trend suggests that OpenAI o3 performs better across all systems, which is an encouraging result supporting its potential use in clinical decision-support roles across various subspecialties.

When comparing the two AI tools in their ability to maintain the same responses after two weeks, the results were unexpected. The results showed that GPT-3.5 has a reproducibility rate of 81.6% compared to 76.7% for OpenAI o3. This change was significant between the two rounds. Moreover, the accuracy of OpenAI o3’s answers dropped from 83.3% to 70%, as the program changed 14 answers the second time around. It corrected 3 answers, while changing the answer to 11 questions from correct to incorrect. In contrast, GPT-3.5 showed better reproducibility, as it maintained an accuracy of approximately 72%, which is close to the first result of 70%.

Our findings aligned with those of Al-Dujaili et al., who evaluated ChatGPT’s precision and reliability in handling pharmaceutical situations. The study found an accuracy rate of 70.83% at week one, 79.2% at week three, and 75% at week five. This suggests that there is significant heterogeneity in accuracy rates at different time intervals [18].

Although OpenAI o3 initially achieved higher accuracy, it showed a notable decline in performance on repeat testing, dropping from 83.3% to 70.0%. This variation raises concerns about consistency, which is essential for clinical decision-making. The change may be related to the model’s internal variability in generating responses or possibly due to updates made by the developers during the study period. Since the exact reason cannot be confirmed, both possibilities should be considered. These findings reinforce the importance of careful and supervised use of such tools in clinical settings.

Overall, these results indicate that advanced AI systems, like OpenAI o3, could serve as clinical decision-support tools. The substantially improved accuracy and reduced error rate suggest the benefits of such tools extend to clinical workflows, as they could verify human decisions and reduce the cognitive burden for pharmacists—especially when handling large volumes of information or complexity—with potential positive impacts on patient safety and system efficiency. It is important to note, however, that despite their potential, AI systems cannot yet substitute the work of a certified clinical pharmacist, particularly for complicated therapeutic decisions.

This study has several limitations that should be acknowledged. First, the study evaluated the performance of only two AI tools (GPT-3.5 and OpenAI o3), which might not accurately represent the performance of all AI systems as clinical decision-support tools. Also, this study included a limited dataset of 60 MCQs. This limited number of questions inherently restricts the scope of the performance evaluation. Moreover, the pharmacists’ responses were collected via an online survey, which may have introduced uncontrolled external influences (e.g., distractions, access to unreported resources, or varying levels of focus), potentially affecting the reliability of their answers.

Importantly, the pharmacist sample was small (*n* = 25), recruited via convenience sampling, and primarily based in Amman. This significantly limits the generalizability of findings even within Jordanian clinical pharmacy settings. Although the participants were all licensed clinical pharmacists (PharmD or MSc), the sample was disproportionately composed of academic preceptors (76%) with fewer hospital-based practitioners (24%), which introduces professional practice bias. Additionally, the sample was skewed with 92% female participants, resulting in a gender imbalance that may limit generalizability due to potential gender-linked variations in experience or clinical roles. This sampling strategy also introduces selection bias.

Furthermore, the “None” option used in the MCQs, although justified in the Methods, is an unusual design feature and may have affected participants’ interpretation of questions. Another limitation is that all AI assessments were conducted in English, which may limit the applicability of the findings in non-English-speaking or multilingual healthcare settings. Additionally, drug–drug interactions (DDIs) were underrepresented, with only three related questions. This limits the study’s ability to assess AI performance in this key area of clinical practice. Moreover, the exclusive focus on pharmacy clinical cases limits the generalizability of the findings to other academic fields. Additionally, although OpenAI o3 demonstrated higher accuracy, it remains unclear whether its correct answers reflect sound clinical reasoning, as the model lacks true contextual understanding and clinical judgment. An extra limitation arises from the dynamic nature of AI models like GPT-3.5 and OpenAI o3, which are frequently updated by their developers. Consequently, the performance observed in this study may change over time, reducing the reproducibility and long-term applicability of our results.

## 5. Conclusions

This study compared the accuracy, sensitivity, specificity, and reproducibility of the free GPT-3.5 and subscription-based OpenAI o3 in answering 60 clinical MCQs, alongside the performance of clinical pharmacists. OpenAI o3 demonstrated superior accuracy, sensitivity, and specificity compared to both GPT-3.5 and the pharmacists. However, GPT-3.5 showed greater reliability and stability, with more consistent results across two assessments conducted two weeks apart. The difficulty level of the questions significantly impacted AI accuracy. Therefore, while advanced AI tools like OpenAI o3 show great promise, their use should be approached with caution, especially given ongoing updates that may influence their performance. The study also emphasizes the importance of ongoing evolution in these platforms to have higher specificity and accuracy and better adapt to real-world clinical scenarios. In the future, combining these advanced AI technologies with established healthcare methods may help create a system where AI supports conventional medical practices, resulting in more tailored, efficient, and safer healthcare delivery.

## Figures and Tables

**Table 1:** MCQs revised based on pilot feedback.

MCQ ID	Feedback Summary	Revised Action Taken
All questions	Add “What is your recommendation?”	Reworded all questions for clarity
2	Edit dose of Candesartan & clarify the question	Reworded question and dosage clarification added
3	Clarify that patient is controlled on HTN medications	Reworded question to reflect patient control
4	Assume adherence to medications	Reworded question to indicate adherence
6	Add “to decrease the possibility of hypotension”	Statement added; question reworded for clarity
16	Add NYHA Class III	Heart failure classification added
19	Add “all postprandial levels are normal”	Postprandial status added; question reworded
46	Specify UTI as “lower cystitis”	Type of UTI specified in question stem
47	Specify UTI as “acute pyelonephritis”	Diagnosis added to clarify UTI type
50	Clarify penicillin allergy as type I anaphylaxis	Allergy type added to exclude incorrect option
52	Remove “Clostridium difficile” from Option D	Revised Option D for accuracy
60	Clarify “torsades de pointes” as “irregular heartbeats”	Revised Option D for clearer understanding

**Table 2:** Top three easiest and hardest MCQs based on difficulty index.

Question No.	Question Text	Correct Answer	DI	Classification
3	SE is a 37-year-old woman patient with known history of Hypertension, which is controlled by Candesartan 16 mg once daily, now she is pregnant. As a clinical pharmacist, what is your recommendation? Switch Candesartan to Telmisartan. Switch Candesartan to Enalapril. Keep on Candesartan since it is safe during pregnancy. Switch Candesartan to Ramipril.	None	1	Easiest
19	SS is a 23-year-old female patient with known history of Type 1 Diabetes mellitus; she is on Glargine 20 unit once daily at bedtime and Insulin Aspart 10/15/10 units before each meal. The patient measures her blood glucose level, and all postprandial levels are normal. However, fasting levels are 220 mg/dl. As a clinical pharmacist, what would you recommend? Increase the dose of Insulin Aspart before breakfast. Increase the dose of Glargine by 2 units. Increase the dose of Insulin Aspart before dinner. Decrease the dose of Glargine by 2 units.	B	1	Easiest
58	SS is a 55-year-old female patient with known history of Chronic heart failure. She is on Enalapril 10 mg twice daily, Carvedilol 12.5 mg twice daily, Furosemide 40 mg twice daily and Ivabradine 5 mg twice daily. She diagnosed as recurrent vulvovaginal candidiasis; the physician starts her on Fluconazole 200 mg orally every 72 h for 14 days. As a clinical pharmacist, what is your recommendation? There is contraindication of concomitant use of Fluconazole and Enalapril. There is contraindication of concomitant use of Fluconazole and Carvedilol. There is contraindication of concomitant use of Fluconazole and Furosemide. There is contraindication of concomitant use of Fluconazole and Ivabradine.	D	1	Easiest
37	AA is a 27-year-old female patient with known history of mild persistent asthma. She is on low dose Beclomethasone and Formoterol (Foster^®^ pMDI). As a clinical pharmacist, what is the most important tip to educate her about her device? Take forceful, deep breathing. Use spacer to decrease side effects. Store the device at room temperature. It isn’t needed for coordination (pressing and breathing).	B	0	Hardest
56	A 33-year-old female patient diagnosed as primary syphilis. As a clinical pharmacist, what is the treatment of choice to be started? Amoxicillin-Clavulanic acid 1 g twice daily for 2 weeks. Ciprofloxacin 500 mg twice daily for 2 weeks. Clindamycin 300 mg twice daily for 2 weeks. Vancomycin 1 g twice daily for 2 weeks.	None	0	Hardest
49	SS is a 33-year-old female patient with penicillin allergy. She was diagnosed as uncomplicated Cystitis. The physician starts her on TMP-SMX, As a clinical pharmacist, which of the following is the most important tip to be educated regarding this medication? It can cause photosensitivity; you should use sunscreen. It can cause QT prolongation. It can chelate metals, so that it is contraindicated to be given with them. It can cause Crystalluria, so that you should drink a lot of water.	D	0.2	Hardest

**Table 3:** Comparison of diagnostic performance metrics between clinical pharmacists and AI tools (GPT-3.5 and OpenAI o3).

Metric	Group Compared	Mean (Pharmacists)	AI Tool Value	*p*-Value
Accuracy	Pharmacists vs. GPT-3.5	69.7%	70.0%	0.852 ^
	Pharmacists vs. OpenAI o3	69.7%	83.3%	<0.001 ^
Sensitivity	Pharmacists vs. GPT-3.5	77.0%	77.5%	0.718 ^
	Pharmacists vs. OpenAI o3	77.0%	90.0%	<0.001 ^
Specificity	Pharmacists vs. GPT-3.5	55.0%	55.0%	0.965 #
	Pharmacists vs. OpenAI o3	55.0%	70.0%	0.001 #

^: One-Sample *t*-test. #: One-Sample Wilcoxon Signed-Rank Test.

**Table 4:** Comparative performance of AI tools across difficulty levels.

AI Tool	Answer Type	Easy (DI > 0.8)	Average (DI > 0.4–0.8)	Difficult (DI 0–0.4)	*p*-Value #
GPT-3.5 (Free model)	Correct	14 (87.5%)	22 (78.6%)	6 (37.5%)	0.007
	Incorrect	2 (12.5%)	6 (21.4%)	10 (62.5%)	
OpenAI o3 (Subscription-based model)	Correct	15 (93.8%)	26 (92.9%)	15 (56.3%)	0.006
	Incorrect	1 (6.3%)	2 (7.1%)	7 (43.8%)	

#: Using Chi-square test or Fisher Exact test.

**Table 5:** Comparative performance of AI tools across therapeutic systems.

AI Tool	Answer Type	Cardiovascular	Endocrine	Respiratory	Infectious Diseases	*p*-Value #
GPT-3.5 (Free model)	Correct	9 (60.0%)	10 (66.7%)	11 (73.3%)	12 (80.0%)	0.782
	Incorrect	6 (40.0%)	5 (33.3%)	4 (26.7%)	3 (20.0%)	
OpenAI o3 (Subscription-based model)	Correct	14 (93.3%)	10 (66.7%)	13 (86.7%)	13 (86.7%)	0.357
	Incorrect	1 (6.7%)	5 (33.3%)	2 (13.3%)	2 (13.3%)	

#: Using Fisher Exact test.

**Table 6:** MCQ questions with AI model disagreement and pharmacist benchmarking.

MCQ ID and Therapeutic System	Question Difficulty Index	Correct Answer	GPT-3.5 (Free Version)	OpenAI o3 (Subscription-Based)	Pharmacists Majority Answer (%)
Q4 CV	Difficult	B	None (FN) 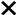	B (TP) 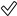	B (72%) 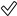
Q12 CV	Easy	D	None (FN) 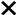	D (TP) 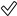	D (96%) 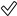
Q13 CV	Easy	A	A (TP) 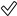	C (FN) 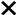	A (56%) 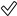
Q20 EN	Difficult	A	C (FN) 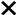	A (TP) 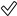	A (64%) 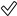
Q25 EN	Easy	None	B (FP) 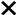	None (TN) 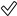	None (56%) 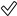
Q39 RE	Easy	A	C (FN) 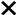	A (TP) 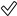	A (56%) 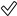
Q40 RE	Difficult	None	None (TN) 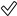	A (FP) 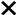	None (40%) 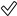
Q48 IN	Difficult	None	D (FP) 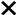	None (TN) 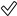	D (52%) 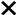
Q50 IN	Difficult	None	D (FP) 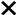	None (TN) 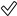	D (28%) 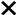
					B (28%) 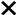
Q53 IN	Easy	A	B (FN) 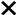	A (TP) 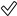	A (80%) 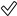
Q54 IN	Difficult	None	B (FP) 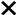	None (TN) 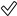	None (32%) 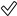
					B (32%)
Q57 IN	Easy	D	A (FN) 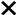	D (TP) 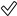	D (64%) 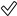
Q58 N	Easy	D	None (FN) 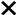	D (TP) 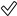	D (84%) 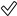
Q59 IN	Difficult	A	A (TP) 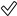	None (FN) 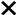	A (48%) 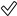

CV: Cardiovascular, EN: Endocrine, RE: Respiratory, IN: Infectious Diseases, 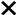: Incorrect Answer, 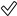: Correct Answer.

**Table 7:** Reproducibility between rounds.

AI Tool	Identical Answers (N)	Total Questions	Reproducibility Index (%)	Cohen’s Kappa	*p*-Value #
GPT-3.5 (Free model)	49	60	81.6	0.556	<0.001
OpenAI o3 (Subscription-based model)	46	60	76.7	0.364	0.002

#: Using Cohen’s Kappa test.

**Table 8:** Evaluating accuracy of AI Tools in multiple test runs.

Model	Correct (1st Run)	Accuracy (1st Run)	Correct (2nd Run)	Accuracy (2nd Run)	Correct Incorrect	Incorrect Correct	*p*-Value #
GPT-3.5 (Free model)	42	70.0%	43	71.6%	5	6	1.000
OpenAI o3 (Subscription-based model)	50	83.3%	42	70.0%	11	3	0.057

#: Using McNemar test.

## Data Availability

The data that support the findings of this study are available from [Rana Abu-Farha] upon reasonable request due to privacy/ethical restrictions.

## Abbreviations

The following abbreviations are used in this manuscript:

MCQs	Multiple-Choice Questions
DI	Difficulty Index
TP	True Positive
TN	True Negative
FP	False Positive
FN	False Negative

## References

[1] Elenbaas R.M., Worthen D.B. (2009). Transformation of a profession: An overview of the 20th century. Pharm. Hist..

[2] Islam R., Weir C., Del Fiol G. (2016). Clinical complexity in medicine: A measurement model of task and patient complexity. Methods Inf. Med..

[3] Raza M.A., Aziz S., Noreen M., Saeed A., Anjum I., Ahmed M., Raza S.M. (2022). Artificial intelligence (AI) in pharmacy: An overview of innovations. Innov. Pharm..

[4] Haleem A., Javaid M., Singh R.P. (2022). An era of ChatGPT as a significant futuristic support tool: A study on features, abilities, and challenges. BenchCouncil Trans. Benchmarks Stand. Eval..

[5] Wiens J., Shenoy E.S. (2018). Machine learning for healthcare: On the verge of a major shift in healthcare epidemiology. Clin. Infect. Dis..

[6] Roosan D., Padua P., Khan R., Khan H., Verzosa C., Wu Y. (2024). Effectiveness of ChatGPT in clinical pharmacy and the role of artificial intelligence in medication therapy management. J. Am. Pharm. Assoc..

[7] Al-Dujaili Z., Hallit S., Al Faraj A. (2023). Knowledge, attitude, and readiness of pharmacists toward medication therapy management for patients with attention deficit hyperactivity disorder: A cross-sectional quantitative study. Int. J. Clin. Pharm..

[8] Tan S., Xin X., Wu D. (2024). ChatGPT in medicine: Prospects and challenges: A review article. Int. J. Surg..

[9] Cortes D., Leung J., Ryl A., Lieu J. (2019). Pharmacy informatics: Where medication use and technology meet. Can. J. Hosp. Pharm..

[10] Magrabi F., Ammenwerth E., McNair J.B., De Keizer N.F., Hyppönen H., Nykänen P., Rigby M., Scott P.J., Vehko T., Wong Z.S.-Y. (2019). Artificial intelligence in clinical decision support: Challenges for evaluating AI and practical implications. Yearb. Med. Inform..

[11] Joksimovic S., Ifenthaler D., Marrone R., De Laat M., Siemens G. (2023). Opportunities of artificial intelligence for supporting complex problem-solving: Findings from a scoping review. Comput. Educ. Artif. Intell..

[12] Tekkeşin A.İ. (2019). Artificial intelligence in healthcare: Past, present and future. Anatol. J. Cardiol..

[13] Lertsakulbunlue S., Kantiwong A. (2024). Development and validation of immediate self-feedback very short answer questions for medical students: Practical implementation of generalizability theory to estimate reliability in formative examination designs. BMC Med. Educ..

[14] Maleki N., Padmanabhan B., Dutta K. AI hallucinations: A misnomer worth clarifying. Proceedings of the 2024 IEEE Conference on Artificial Intelligence (CAI).

[15] Albogami Y., Alfakhri A., Alaqil A., Alkoraishi A., Alshammari H., Elsharawy Y., Alhammad A., Alhossan A. (2024). Safety and quality of AI chatbots for drug-related inquiries: A real-world comparison with licensed pharmacists. Digit. Health.

[16] Wang Y.-M., Shen H.-W., Chen T.-J., Chiang S.-C., Lin T.-G. (2025). Performance of ChatGPT-3.5 and ChatGPT-4 in the Taiwan National Pharmacist Licensing Examination: Comparative Evaluation Study. JMIR Med. Educ..

[17] Taesotikul S., Singhan W., Taesotikul T. (2024). ChatGPT vs pharmacy students in the pharmacotherapy time-limit test: A comparative study in Thailand. Curr. Pharm. Teach. Learn..

[18] Al-Dujaili Z., Omari S., Pillai J., Al Faraj A. (2023). Assessing the accuracy and consistency of ChatGPT in clinical pharmacy management: A preliminary analysis with clinical pharmacy experts worldwide. Res. Social. Adm. Pharm..

